# Resilient by Design: Environmental Stress Promotes Biofilm Formation and Multi-Resistance in Poultry-Associated *Salmonella*

**DOI:** 10.3390/microorganisms13081812

**Published:** 2025-08-03

**Authors:** Gabriel I. Krüger, Francisca Urbina, Coral Pardo-Esté, Valentina Salinas, Javiera Álvarez, Nicolás Avilés, Ana Oviedo, Catalina Kusch, Valentina Pavez, Rolando Vernal, Mario Tello, Luis Alvarez-Thon, Juan Castro-Severyn, Francisco Remonsellez, Alejandro Hidalgo, Claudia P. Saavedra

**Affiliations:** 1 Laboratorio de Microbiología Molecular, Facultad de Ciencias de la Vida, Universidad Andres Bello, Republica 330, Santiago 8370146, Chile; g.krugercarrasco@gmail.com (G.I.K.); fran.urbina17@gmail.com (F.U.); cpardoeste@gmail.com (C.P.-E.); valesalinasb@gmail.com (V.S.); jalvarezm1011@gmail.com (J.Á.); nicolas.aviles0170@gmail.com (N.A.); acob11doctorado@gmail.com (A.O.); ckusch03@gmail.com (C.K.); v.pavez.03@gmail.com (V.P.); jsevereyn@gmail.com (J.C.-S.); 2 Laboratorio de Microbiología Aplicada y Extremófilos, Departamento de Ingeniería Química, Universidad Católica del Norte, Antofagasta 1270709, Chile; fremonse@ucn.cl; 3 Laboratorio de Ecología Molecular y Microbiología Aplicada, Departamento de Ciencias Farmacéuticas, Facultad de Ciencias, Universidad Católica del Norte, Antofagasta 1270709, Chile; 4 Allergic Inflammation Group, Department of Immunology and Microbiology, University of Copenhagen, DK-1165 Copenhagen, Denmark; 5 Laboratorio de Biología Periodontal, Facultad de Odontología, Universidad de Chile, Santiago 8330015, Chile; rvernal@uchile.cl; 6 Laboratorio de Metagenómica Bacteriana, Centro de Biotecnología Acuícola, Departamento de Biología, Facultad de Química y Biología, Universidad de Santiago de Chile, Santiago 9170124, Chile; mario.tello@usach.cl; 7 Centro de Investigación en Ingeniería de Materiales (CIIMAT), Facultad de Ingeniería y Arquitectura, Universidad Central, Santiago 8320000, Chile; luis.alvarez@ucentral.cl; 8 Escuela de Química y Farmacia, Facultad de Medicina, Universidad Andres Bello, Santiago 8370146, Chile; alejandro.a.hidalgo@gmail.com

**Keywords:** *Salmonella*, poultry farm, biofilm, antimicrobial resistance, environmental stress

## Abstract

*Salmonella* is one of the main causes of food-borne illness worldwide. In most cases, *Salmonella* contamination can be traced back to food processing plants and/or to cross-contamination during food preparation. To avoid food-borne diseases, food processing plants use sanitizers and biocidal to reduce bacterial contaminants below acceptable levels. Despite these preventive actions, *Salmonella* can survive and consequently affect human health. This study investigates the adaptive capacity of the main *Salmonella enterica* serotypes isolated from the poultry production line, focusing on their replication, antimicrobial resistance, and biofilm formation under stressors such as acidic conditions, oxidative environment, and high osmolarity. Using growth curve analysis, crystal violet staining, and microscopy, we assessed replication, biofilm formation, and antimicrobial resistance under acidic, oxidative, and osmotic stress conditions. Disinfectant tolerance was evaluated by determining the Minimum Inhibitory Concentration (MIC) and Minimum Bactericidal Concentration (MBC) of sodium hypochlorite. The antibiotic resistance was assessed using the Kirby–Bauer method. The results indicate that, in general, acidic and osmotic stress reduce the growth of *Salmonella*. However, no significant differences were observed specifically for serotypes Infantis, Heidelberg, and Corvallis. The *S.* Infantis isolates were the strongest biofilm producers and showed the highest prevalence of multidrug resistance (71%). Interestingly, *S.* Infantis forming biofilms required up to 8-fold higher concentrations of sodium hypochlorite for eradication. Furthermore, osmotic and oxidative stress significantly induced biofilm production in industrial *S.* Infantis isolates compared to a reference strain. Understanding how *Salmonella* responds to industrial stressors is vital for designing strategies to control the proliferation of these highly adapted, multi-resistant pathogens.

## 1. Introduction

The high prevalence of *Salmonella* in poultry production lines represents a challenge to global food security [1]. *Salmonella* is known for its capacity to adapt and survive in highly stressful environments [2], allowing *Salmonella* to remain in the food production line of poultry meat, despite rigorous cleaning and disinfection procedures [3]. This phenomenon is particularly concerning in the context of the poultry industry, where *Salmonella* strains have shown increasing resistance [4,5].

Previous studies have demonstrated the resistance of these bacteria to different stressors [6]. Acidic, oxidative, and osmotic stress are among the most common in the production line [6,7,8]. Thus, antimicrobial resistance, including resistance to disinfectants and antibiotics, is a recurrent threat to food safety and public health. These bacteria are constantly subjected to environmental challenges during the poultry production process, including pH variations, reduced water activity, and low antimicrobial concentrations, which can exert selective pressure [9]. These conditions can also promote adaptation, allowing bacteria to grow and survive under such challenges [10]. Even though strict cleaning and sanitation protocols are applied in the production line, pathogens can persist on surfaces, a phenomenon linked to their biofilm-forming capacity [11], which enhances their overall resistance to environmental abrasion and stressors. The ability to form biofilms has been associated with both resistance and the development of tolerance to environmental stress [12,13]. Moreover, the high cell density within a biofilm and bacterial proximity facilitate horizontal gene transfer [14,15]. It is crucial to consider these innate and acquired factors, as some pathogens with adaptive capacity may use similar mechanisms to develop antibiotic resistance via protective effects or cross-resistance [16].

Recent investigations have shown that sublethal doses of antimicrobials and biocides can induce biofilm production by *Salmonella* [17]. These antimicrobials, including sodium hypochlorite and benzalkonium chloride, may select for more resistant subpopulations capable of forming tighter biofilms, thus increasing the difficulty of eradicating these pathogens from food processing facilities [18]. Biofilm formation by *Salmonella* is significantly modulated by environmental stimuli, particularly those present in the poultry production line. Biofilm formation is regulated by several genes, among which the transcriptional regulator *csgD* and the *csg* cluster are crucial for biofilm formation under variable environmental conditions [19]. Furthermore, significant differences have been observed between *Salmonella* Enteritidis strains from poultry and human sources. *Salmonella* Enteritidis from poultry exhibited great biofilm-forming capacity and higher expression of genes such as *adrA* and *csgD*, responsible for the synthesis of EPS (extracellular polymeric substances) components, and *luxS*, involved in quorum sensing, compared to human-isolated *Salmonella* Enteritidis [20]. These differences suggest that the isolation source of *Salmonella* plays a crucial role in its adaptive capacity in stressful environments, with poultry-derived strains being more prone to developing robust survival mechanisms compared to strains from non-industrial origins.

Despite extensive studies on the capacity of *Salmonella* to withstand environmental stress, many questions remain about its robust response within the poultry industry and how the biofilm-forming capacity and composition influence survival and resistance against oxidative, acidic, and osmotic stressors in the poultry production line. Therefore, the objective of this follow-up study [4,5,21] is to compare *Salmonella* isolates from the poultry production line in terms of antimicrobial resistance and biofilm-forming capacity using a comparative and associative strategy, and the correlation between antimicrobial resistance capacity, biofilm-forming capacity, and EPS composition will also be explored among *Salmonella* isolates.

## 2. Materials and Methods

### 2.1. Study Design

For this study, 155 isolates of *Salmonella enterica* isolated between 2018 and 2021 from a poultry meat production line in Chile were characterized, as previously described [4]. In brief, the isolates were collected from different areas and surfaces across the facility, including the feed mill, the hatchery, the chicken farm, and the slaughterhouse. Sampling, isolation, and confirmation of *Salmonella* were performed as previously described [5]. Additionally, physiological growth and biofilm formation were assessed alongside a *Salmonella* Infantis SARB27 strain (ATCC BAA-1675), which was used as a reference strain and an external control.

### 2.2. Culture Conditions and Growth Under Osmotic and Acid Stress

The acidic and osmotic stress conditions were induced using minimal glucose medium (MgM) as previously described [22]. Briefly, the basal medium contained 7.5 mM (NH_4_)_2_SO_4_ (Merck, Darmstadt, Germany), 5 mM KCl (BioPack, Buenos Aires, Argentina), 0.5 mM K_2_SO_4_ (Winkler, Santiago, Chile), 1 mM KH_2_PO_4_ (J. T. Baker, Radnor, PA, USA), 10 mM MgCl_2_ (Merck, Darmstadt, Germany), 2 mM glucose (ITW Reagents, Monza, Italy), and 0.1% casamino acids (Gibco, Grand Island, NY, USA). For the different conditions, this medium was buffered to pH 5.6 with 100 mM MES (2-(N-morpholino) ethanesulfonic acid; Sigma, Steinheim, Germany) (acid medium) or to pH 7.2 with 100 mM Tris (PhytoTech Labs, Lenexa, KS, USA) (control medium). For the osmotic stress condition, the pH 7.2 control medium was supplemented with 15% (*w*/*v*) sucrose (ITW Reagents, Monza, Italy). To obtain the growth profiles, each *Salmonella* isolate was first grown overnight (16 h) in LB at 37 °C with shaking (150 rpm). A 1:100 aliquot of this starter culture was then inoculated into the control MgM medium (pH 7.2) and incubated for 24 h under the same conditions to create pre-cultures. Afterwards, the pre-cultures were sub-cultured (1:50) into acidic MgM (pH 5.6), osmotic MgM (pH 7.2 + 15% sucrose), and control MgM (pH 7.2) and incubated for 8 h. The optical density at 600 nm (OD_600_) was measured every hour (*n* = 3). The doubling time (Td) was determined by fitting an exponential function to the growth curve: $y=Ae^{Bx}$, where y is the OD_600_ at time x, A is the initial OD_600_, and B is the specific growth rate constant. The doubling time was then calculated as Td=ln2B≈0.693B.

### 2.3. Sodium Hypochlorite Minimal Inhibitory Concentration (MIC) Determination

Each isolate was cultured in 5 mL of Mueller–Hinton broth (MHB) (Gibco, Grand Island, NY, USA) medium for 16 h with shaking at 150 rpm and at 37 °C. After incubation, the cultures were inoculated at a 1:100 ratio in 5 mL of MHB medium, and their growth was monitored until reaching an OD_600_ value between 0.4 and 0.5. Then, an aliquot was diluted in MHB to an OD_600_ of approximately 0.04. Meanwhile, dilutions of sodium hypochlorite (NaClO) (Sigma, Steinheim, Germany) were prepared from a 640 Mm stock solution. The MIC determination was carried out in sterile 96-well plates (Thermo Scientific; St. Louis, MO, USA), where 190 μL of the diluted culture was added and combined with 10 μL of the corresponding NaClO dilution to obtain final concentrations of 32, 16, 8, 4, 2, 1, and 0.5 mM. Each condition was tested using three biological replicates, each with three technical replicates (*n* = 9). Additionally, wells without NaClO served as growth control. After preparation, the plates were incubated for 16 h without shaking at 37 °C. Subsequently, absorbance OD_600_ was measured using an Infinite 200 PRO plate reader (Tecan Life Sciences, Männedorf, Switzerland). The MIC was determined as the lowest concentration of NaClO that prevented bacterial growth.

### 2.4. Biofilm Formation Capacity and Determination of the Intensity in the Presence of Sodium Hypochlorite

Biofilm formation and intensity quantification were determined as described by Stepanović et al. (2007) [23]. Briefly, biofilm mass was quantified using a crystal violet assay in 96-well microplates (Thermo Scientific; St. Louis, MO, USA). Wells were prepared with 190 μL of MHB medium containing 10 μL of NaClO (at half the MIC for each respective isolate) and were then inoculated with approximately 5 × 10^5^ Colony Forming Units (CFU). The plates were then incubated for 24 h without shaking at 37 °C. After incubation, the medium was aspirated, and the wells were washed three times with sterile phosphate-buffered saline (PBS). To fix the biofilms, the plates were dried in an inverted position at 60 °C for 1 h. The fixed biofilms were stained by adding 150 μL of 0.1% crystal violet and incubating them for 15 min at room temperature. Excess stains were removed by washing with PBS. The bound stain was then solubilized by adding 150 μL of 33% acetic acid. Absorbance was measured at 570 nm using an Infinite 200 PRO plate reader (Tecan Life Sciences, Männedorf, Switzerland). For each isolate, biofilm formation and its intensity were assayed in three biological replicates, with five technical replicates per plate (*n* = 15). Biofilm formation was interpreted as follows: the cutoff optical density (ODc) was defined as the mean absorbance of the negative control wells, plus their standard deviation, three times. Isolates were then classified based on their mean absorbance (OD_570nm_):

0. Non-forming isolates: OD_570nm_ ≤ ODc.
1. Week biofilm formation isolate: ODc < OD_570nm_ ≤ 2xODc.
2. Moderate biofilm formation isolate: 2xODc < OD_570nm_ ≤ 3xODc.
3. Strong biofilm formation isolate: 3xODc < OD_570nm_ ≤ 4xODc.
4. Very strong biofilm formation isolate: OD_570nm_ > 4xODc.

### 2.5. Bactericidal and Minimal Inhibitory Concentration of Sodium Hypochlorite in Biofilm

The Minimum Inhibitory Concentration in Biofilm (MICB) and Minimum Bactericidal Concentration (MBCB) of NaClO were determined for isolates that exhibited very strong biofilm formation. The assay was performed on 96-well microtiter plates with peg lids (Thermo Scientific; St. Louis, MO, USA). First, to form biofilms on the pegs, 200 μL of the MHB medium was added to each well and inoculated with approximately 5 × 10^5^ CFU. The plate, covered with the peg lid, was then incubated for 24 h without agitation at 37 °C. Subsequently, a second plate was prepared with serial dilutions of NaClO in MHB (final concentrations of 32, 16, 8, 4, 2, 1, and 0.5 mM). After biofilm incubation, the peg lid was washed three times with PBS to remove non-adherent cells and then transferred to a challenge plate with the NaClO gradient. The plate was incubated for 24 h without agitation at 37 °C. After this period, the absorbance (OD_600_) was measured in the Infinite 200 PRO reader (Tecan Life Sciences, Männedorf, Switzerland) to determine the MICB. To determine the MBCB, the peg lid was then retrieved from the challenge plate, washed three times with PBS, and transferred to a new plate containing only the MHB medium. This recovery plate was incubated for 24 h without agitation at 37 °C. Finally, after this recovery incubation, the OD_600nm_ of the recovery plate was measured. The MBCB was defined as the lowest concentration of NaClO that resulted in no subsequent growth.

### 2.6. Antimicrobial Susceptibility Testing

All isolates were subjected to a panel of 20 antibiotics following the CLSI guidelines [24] for the Enterobacterales group of bacteria using the Kirby–Bauer method in Mueller–Hinton agar plates. The tested antibiotics included ampicillin (AMP, 10 μg); cefazolin (KZ, 30 μg); cefepime (FEP, 30 μg); ceftriaxone (CRO, 30 μg); ceftazidime (CAZ, 30 μg); ciprofloxacin (CIP, 5 μg); gentamicin (CN, 10 μg); amikacin (AMK, 30 μg); imipenem (IPM, 10 μg); meropenem (MEM, 10 μg); azithromycin (AZM, 15 μg); tetracycline (TE, 30 μg); ceftazidime/avibactam (CZA 10/4 μg); piperacillin/tazobactam (TZP, 100/10 μg); trimethoprim/sulfamethoxazole (SXT 1.25/23.75 μg); ampicillin/sulbactam (SAM 10/10 μg); nitrofurantoin (F, 200 μg); chloramphenicol (C, 30 μg); and aztreonam (ATM, 30 μg). All sensidiscs were from OXOID (OXOID, Hampshire, UK). Isolates resistant to three or more classes of antimicrobials were categorized as multidrug-resistant (MDR).

### 2.7. Scanning Electron Microscopy Analysis

Selected bacterial isolates were observed under scanning electron microscopy (SEM). To visualize changes in the biofilm under acid, osmotic, and oxidative stress conditions, the selected isolates were cultured in 24-well plates, with each well containing a 13 mm diameter plastic coverslip (Thermo Scientific; St. Louis, MO, USA). Each well was filled with 750 μL of the respective culture medium inoculated with the selected isolates and then incubated for 24 h without shaking at 37 °C. After incubation, the medium from each well was aspirated, and the coverslips were washed three times with sterile PBS. The biofilms were then fixed by incubating the coverslips in 2.5% glutaraldehyde at 4 °C overnight (16 h). The fixed samples were then prepared and analyzed by scanning electron microscopy using a JOEL SEM model JSM-IT 300 (CIQTEK Co., Hefei, Anhui, China).

### 2.8. Extracellular Polymeric Substances Determination

Extracellular polymeric substances were extracted from the supernatants of *Salmonella* cultures grown under biofilm-forming conditions. Approximately 10 mL of culture was centrifuged (15,000× *g*, 2 h, 4 °C), and the resulting supernatant was pressure-filtered (0.22 µm filter). Next, the EPS were precipitated from the filtered supernatant by adding three volumes of cold absolute ethanol (30 mL), and the mixture was incubated at −20 °C for 24 h. The precipitate was washed twice with ethanol (30 mL), and the mixture was incubated at −20 °C for 24 h. The precipitate was washed twice with ethanol and collected by centrifugation. It was then resuspended in Milli-Q water and purified by dialysis against distilled water at 4 °C. The purified sample was concentrated under vacuum and then lyophilized for 12 h. Their total EPS yield was quantified gravimetrically after lyophilization. Total neutral carbohydrates were measured using phenol-sulfuric acid [25]. Briefly, 100 µg of purified EPS was mixed with 125 µL of concentrated sulfuric acid, followed by the addition of 25 µL of 10% phenol. The mixture was vortexed for 1 min and then incubated at 95 °C for 5 min. After cooling, the samples were transferred to a 96-well plate, and the absorbance at 490 nm was recorded with a Synergy HT spectrophotometer (BioTek, Santa Clara, CA, USA). Concentrations were determined relative to a glucose standard. Protein content was determined by the Bradford method with bovine serum albumin as a standard. The extracellular DNA (eDNA) content in the EPS was quantified by measuring absorbance at 260 nm using a spectrophotometer.

### 2.9. Statistical Analysis

All quantitative data are presented as the mean ± standard deviation (SD). Each experiment was conducted with a minimum of three independent biological replicates; the number of replicates for each specific assay is detailed in the corresponding methods section. Statistical analyses were performed using GraphPad Prism software v.10.5.0 (GraphPad Software, Inc., San Diego, CA, USA). Differences between experimental groups were assessed using one-way or two-way analysis of variance (ANOVA), followed by an appropriate post hoc test for multiple comparisons. A *p*-value < 0.05 was considered statistically significant.

## 3. Results

### 3.1. Variability of Stress Response Performance Profiles in a Salmonella Population from the Poultry Production Line

To evaluate the capacity of isolates to respond to various stress factors within the poultry industry, we assessed their growth performance during acidic, osmotic, and oxidative stress. The experimental design applied different methodologies specific to each stressor, based on its relevance in the poultry production environment. For persistent stressors such as altered pH and high osmolarity, their effects were assessed through growth kinetics by determining changes in doubling time. This approach provided an overview of the stress response capabilities across a diverse set of isolates.

**Figure 1:**
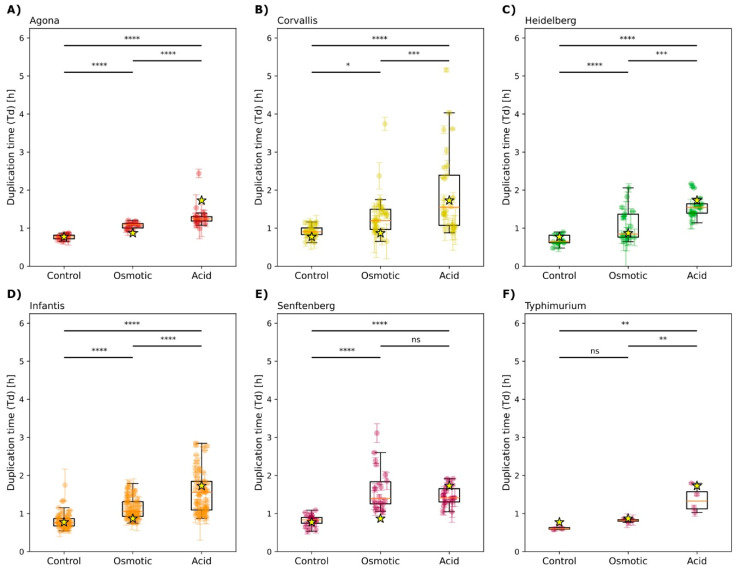
**Doubling times under acidic and osmotic stress.** Osmotic stress was induced by culturing in media supplemented with 15% sucrose, while acidic stress was induced with MES buffer adjusted to pH 5.6. The panels display the doubling times for isolates of (**A**) *Salmonella* Agona; (**B**) *Salmonella* Corvallis; (**C**) *Salmonella* Heidelberg; (**D**) *Salmonella* Infantis; (**E**) *Salmonella* Senftenberg; and (**F**) *Salmonella* Typhimurium. Outliers not included in the statistical analysis are indicated by crosses, and values corresponding to the *Salmonella* Infantis SARB27 strain are indicated by a star. The significance between treatments was determined by two-way ANOVA (ns: not significant, * *p* < 0.05; ** *p* < 0.01; *** *p* < 0.001; **** *p* < 0.0001).

The results demonstrated that the growth performance of the isolates was generally reduced under acidic and osmotic stress, as reflected by increased doubling times. Although the treatment groups showed significant differences compared to the control (Figure 1), most of the isolates of serotypes Infantis, Heidelberg, and Corvallis did not have significant differences in growth when compared to the control (Supplementary Table S1).

In contrast, the response to oxidative stress is much better reflected in the resistance to toxic agents; therefore, it was evaluated by determining the Minimum Inhibitory Concentration (MIC) of sodium hypochlorite against planktonic *Salmonella* and assessing the biofilm formation capacity. Overall, the most frequent MIC value for the isolate was 4 mM sodium hypochlorite (Table 1), while their primary difference was in their biofilm-forming capacity, where both forming and non-forming profiles were exhibited across all serotypes (Table 2). Our analysis of biofilm formation intensity, which correlates with the quantity of biomass, revealed even greater diversity (Table 2). A total of 52 isolates were classified as very strong biofilm formers, with the serotype Infantis accounting for the largest proportion of its isolates within this category.

### 3.2. Differential Resistance of Salmonella Biofilms to Oxidative Stress Induced by Hypochlorite

When the strongest biofilm-forming isolates were subjected to oxidative stress with sodium hypochlorite, they demonstrated considerably increased tolerance to the disinfectant. The MICB doubled in most cases, and the MBCB was four- to eight-fold higher than the respective planktonic MIC values (Table 3). Consistent with other results, *S.* Infantis isolates exhibited the highest resistance, with an MBCB of 32 mM of sodium hypochlorite, followed by *S*. Corvallis and *S*. Typhimurium isolates (MBCB 16 mM). This variability in biofilm resistance suggests a diverse range of tolerance profiles throughout the production line.

### 3.3. Multidrug-Resistance Profiles Are Concentrated in the Salmonella Infantis Serotype

**Figure 2:**
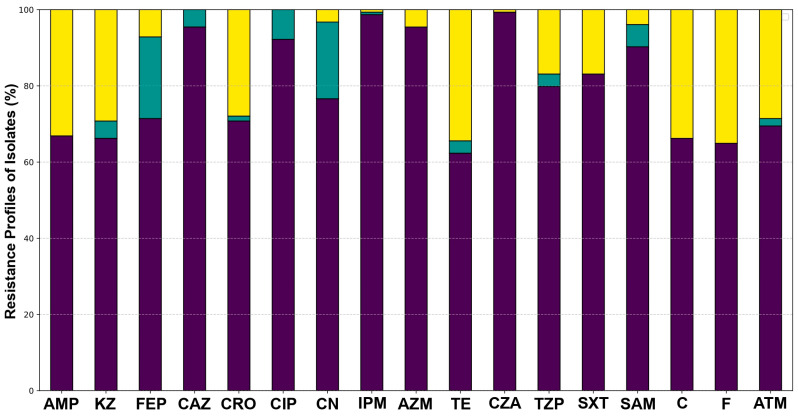
**Antibiotic resistance profiles of *Salmonella* isolates from the poultry production line.** Each vertical bar represents the percentage of *Salmonella* isolates exhibiting different resistance phenotypes against a specific antibiotic. The colored segments within each bar indicate the proportion of isolates classified as: Susceptible in purple, Intermediate in teal, and Resistant in yellow.

The antimicrobial susceptibility of all *Salmonella* isolates from the poultry production line was evaluated against a panel of antibiotics representing different classes. The overall resistance profile of the entire population of isolates is summarized in Figure 2. High percentages of resistance were observed for tetracycline (TE), ampicillin (AMP), chloramphenicol (C), nitrofurantoin (F), and trimethoprim-sulfamethoxazole (SXT). Conversely, high susceptibility (>90% of isolates) was observed for most third- and fourth-generation cephalosporins, such as ceftazidime (CAZ), ceftriaxone (CRO), and cefepime (FEP), as well as for carbapenems (IPM) and β-lactam/β-lactamase inhibitor combinations. Detailed susceptibility profiles for each isolate are provided in Supplementary Table S2.

**Figure 3:**
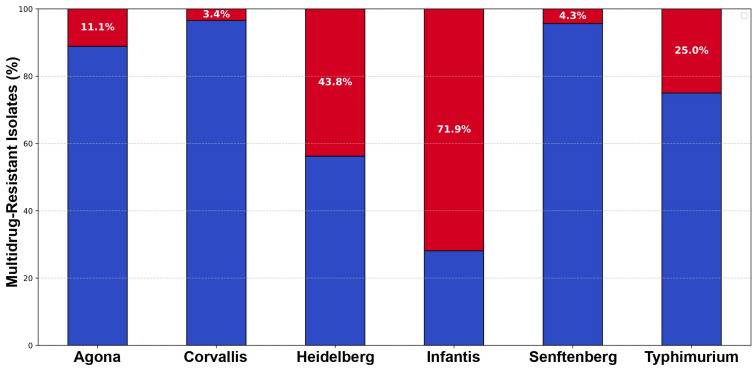
**Frequency of multidrug-resistance profiles among the serotypes identified in the poultry production line.** Prevalence of MDR among the six *Salmonella* serotypes identified in the study is shown. Each bar corresponds to a specific serotype and represents 100% of its isolates. The red section of each bar indicates the percentage of isolates from that serotype that were classified as MDR, while the blue section represents the non-MDR isolates.

Based on these profiles, the prevalence of MDR was determined. Overall, 37.4% (58/155 isolates) were classified as MDR, whereas the remaining 62.7% (97/155 isolates) were not. Analysis of the distribution of this phenotype among the identified serotypes revealed notable differences (Figure 3).

*S.* Infantis was the serotype with the highest frequency of MDR profiles, with 71.9% of its isolates (46/64) exhibiting this characteristic. It was followed by *S.* Heidelberg with 43.8% of its isolates classified as MDR (7/16) and *S.* Agona with 11.1% (2/18). In contrast, each of the remaining serotypes had only a single MDR isolate, as follows: *S.* Corvallis (3.4%; 1/29), *S.* Senftenberg (4.3%; 1/23), and *S.* Typhimurium (25%; 1/4).

### 3.4. Osmotic Stress Induces Enhanced Biofilm Formation in Salmonella Infantis Isolates from the Poultry Production Line

To further investigate the mechanisms driving the persistence and spread of these isolates within the poultry production environment, biofilm formation was studied as a key adaptive process. For this analysis, two representative isolates from critical points in the production line were selected: isolate SE016, recovered from the poultry farm environment, and isolate SE081, obtained from surfaces in the slaughterhouse production line, both of the *S*. Infantis serotype. These strains were selected based on their notable performance against various stress factors and on specific genomic and physiological characteristics previously described by our group; for instance, both carry the pESI-like plasmid that confers the MDR profile and enhances virulence [4,21].

**Figure 4:**
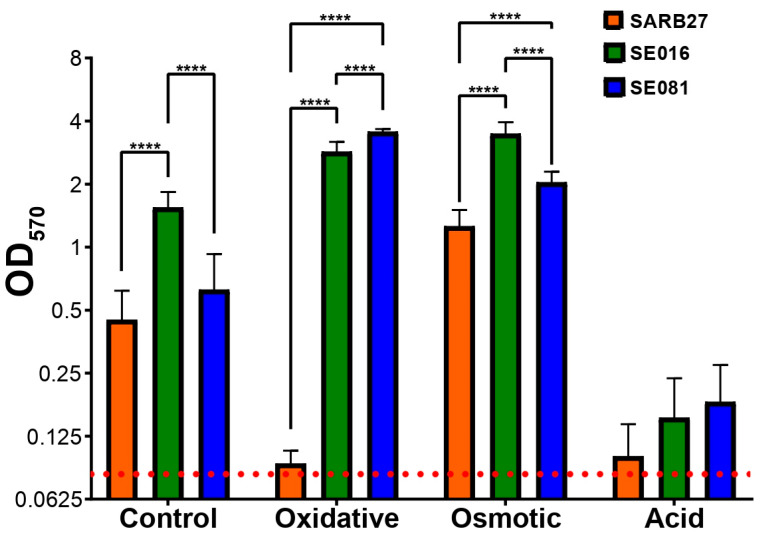
Biofilm formation capacity of isolates SE016 (poultry farm) and SE081 (slaughterhouse) under different stress conditions: oxidative, osmotic, and acid. Biofilm formation capacity, represented on the *Y*-axis, was quantified by normalizing the optical density of the crystal violet-stained biomass by the total bacterial growth (OD_570_). The dotted red line represents the threshold for the negative control. Strain SARB27 was used as a control external to the poultry production line. The significance between treatments was determined by one-way ANOVA (ns: not significant; **** *p* < 0.0001). Not significant values were not added.

The ability of the selected isolates SE016 and SE081, along with the control strain SARB27, to form biofilms under stress conditions relevant to the poultry industry was evaluated. Under control (non-stress) conditions, all three strains demonstrated biofilm-forming capacity, with isolate SE016 producing the highest levels of biofilm (Figure 4). Notably, exposure to either oxidative or osmotic stress significantly increased the biofilm-forming capacity of the industrial isolates SE016 and SE081, which subsequently produced the highest levels of biomass. Specifically, isolate SE081 showed the greatest biofilm formation under oxidative conditions, while isolate SE016 displayed the highest capacity under osmotic stress. In contrast, under acidic stress conditions, both the poultry isolates and the control strain SARB27 exhibited a drastic reduction in biofilm formation, with no significant differences observed among them.

**Figure 5:**
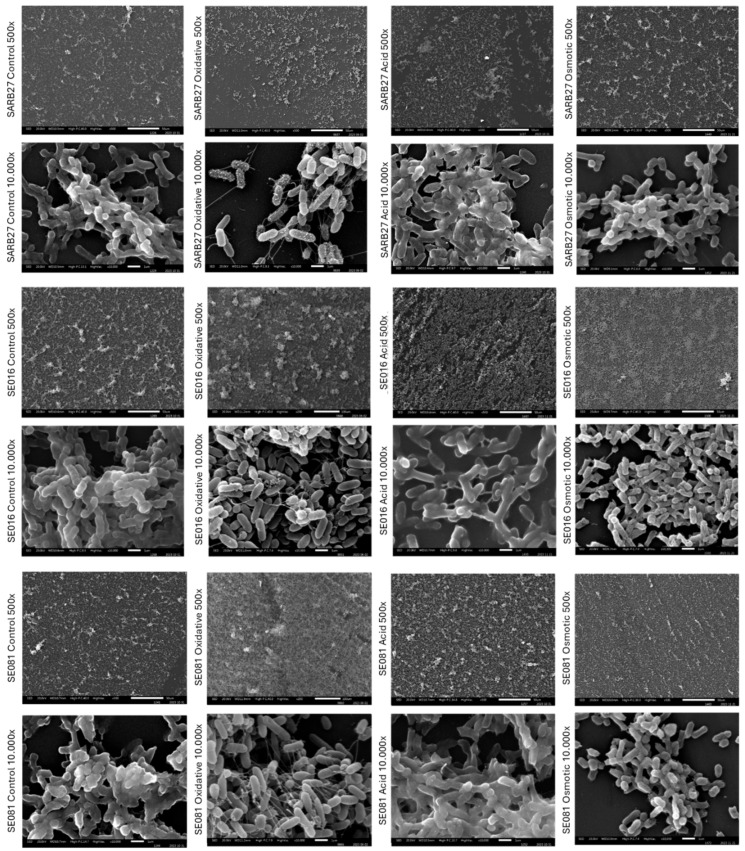
**Analysis of biofilm architecture by scanning electron microscopy (SEM).** Representative micrographs show the structure of biofilms formed by the *Salmonella* Infantis strains SARB27 (control), SE016, and SE081. Biofilms were grown under oxidative, acidic, and osmotic stress, and compared with control condition.

To validate these quantitative findings and analyze biofilm architecture, we performed scanning electron microscopy (SEM) analysis (Figure 5). The micrographs were consistent with the quantification results. Under osmotic and oxidative stress, the isolates SE016 and SE081 formed dense biofilms characterized by high surface occupancy and the presence of three-dimensional aggregates of cells embedded in the extracellular matrix. In contrast, under acidic stress conditions, low-magnification (500×) images showed a sparsely populated surface compared to the control conditions. At higher magnification (10,000×), only dispersed clusters and isolated cells were observed (for SE016 and SARB27), lacking an organized biofilm structure.

### 3.5. Osmotic Stress Enhances the Production of a Polysaccharide-Rich EPS Matrix

Among the many physicochemical challenges that bacteria face in the poultry industry, data from our study highlighted osmotic stress as a condition of relevance. This type of stress, generated by high salt concentrations in processes such as brining, the action of certain disinfectants, and low water activity on surfaces, has been described as a factor that not only selects for more resistant strains but can also enhance adhesion and biofilm formation in *Salmonella* [26,27].

**Figure 6:**
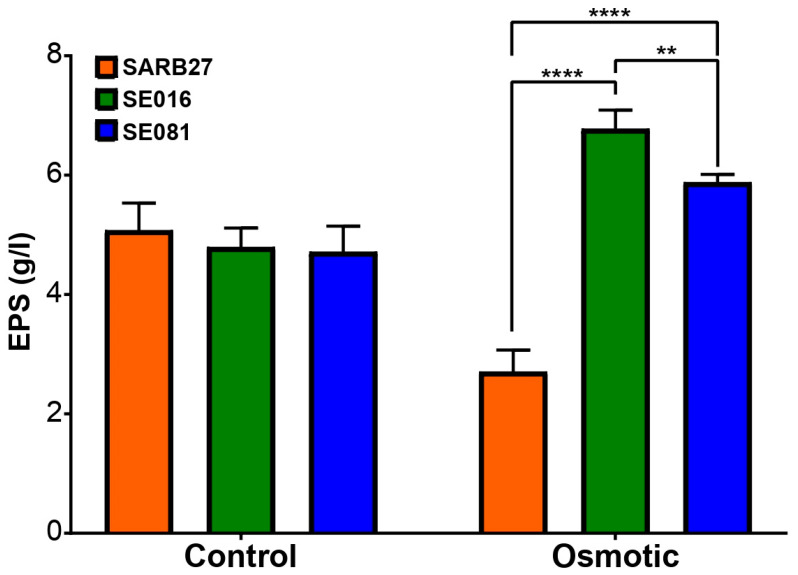
**Quantification of EPS under osmotic stress.** Comparison of the production of EPS, expressed in grams per liter (g/L), by *Salmonella* Infantis strains SARB27, SE016, and SE081. Quantification was performed under the influence of both osmotic stress and control conditions. The significance between treatments was determined by one-way ANOVA (ns: not significant; ** *p* < 0.01; **** *p* < 0.0001). Not significant values were not added.

Given that the EPS matrix is the primary structural component of biofilms, its production was quantified, and its composition was analyzed. The decision to further investigate EPS production only under osmotic stress was strategic and data-driven. Acidic stress was excluded because it severely inhibited biofilm development. The analysis of EPS production under osmotic stress revealed strain-dependent responses (Figure 6). Specifically, under osmotic stress, isolates SE016 and SE081 showed a significant increase in EPS production compared to both their control condition and to the reference strain SARB27. This increase was particularly notable in isolate SE016.

**Figure 7:**
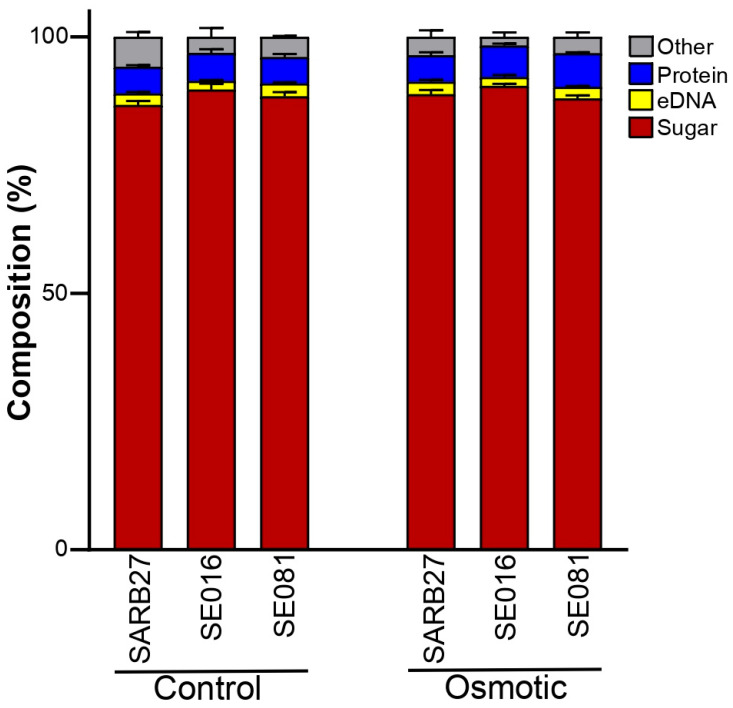
**Composition of the EPS.** The relative composition of the EPS matrix produced by *Salmonella* Infantis strains SARB27, SE016, and SE081 under both control and osmotic stress conditions. The quantified components were sugars (polysaccharides), extracellular DNA (eDNA), and proteins.

Subsequently, the biochemical composition of the EPS matrix was analyzed to determine whether the stress modified its main components (Figure 7). The analysis revealed that, for all strains and under both conditions, sugars (polysaccharides) were the major component, accounting for approximately 90% of the total matrix. Despite the marked differences in the total of EPS produced, statistical analysis did not detect significant changes in the relative percentage composition of the main components in response to osmotic stress. However, it is noteworthy that under osmotic stress, isolate SE016 produced a matrix containing a significantly greater proportion of polysaccharides compared to isolate SE081 (*p* < 0.01). These results highlight the importance of biofilm formation in the response to osmotic stress present in the production line of the poultry industry.

Although both oxidative and osmotic stress enhanced biofilm biomass, osmotic stress was selected for further analysis due to its specific ecological relevance. This condition not only mimics common industrial situations, such as surface desiccation, but was also identified in this study as a strong inducer of EPS production.

## 4. Discussion

The prevalence of *Salmonella* in the poultry production environment constitutes a persistent challenge for food safety [1]. Despite the implementation of extensive cleaning and disinfection protocols, the prevalence of this pathogen remains high, suggesting the involvement of key adaptive mechanisms that allow it to survive, thrive, and spread in hostile industrial environments [28]. This study focused on unraveling the underlying physiological, structural, and population-level mechanisms that enable *Salmonella* permanence. Our findings, based on the analysis of a diverse collection of 155 *Salmonella* isolates recovered from throughout the entire poultry production line, revealed that biofilm formation is not merely a survival mechanism but the central strategy that underpins resistance to multiple stressors, facilitating its prevalence within the production line. This conclusion aligns with other studies that establish that biofilm formation is the predominant lifestyle of bacteria in industrial settings [29,30]. These sessile communities, embedded in an EPS matrix, offer robust protection against a broad spectrum of stressors, including disinfectants, pH fluctuations, osmotic stress, oxidative stress, and even antibiotics [29].

Our data suggests that the poultry production line should not be viewed solely as a passive contamination route, but rather as an active evolutionary scenario that exerts intense selective pressure. The production process, from feed manufacturing and the hatchery to the broiler farm and the slaughterhouse, exposes bacteria to a programmed sequence of distinct stressors, such as nutritional limitations, thermal variations, antibiotic exposure, and an intensive physicochemical assault at the processing plant [31]. Our results demonstrate a clear hierarchy of biological fitness among the serotypes, where *S.* Infantis consistently emerges as the top performer, exhibiting greater stress tolerance, a superior biofilm-forming capacity, more robust antibiotic resistance profiles, and the least impacted cellular doubling time.

The ability of a bacterial population to adapt to changing environments depends fundamentally on its phenotypic diversity [32]. Our results highlight the heterogeneity of the *Salmonella* population, as demonstrated by the variability in the doubling time of the different serotypes under acidic (pH 5.6) and osmotic (15% sucrose) stress conditions (Figure 1). While the growth of most isolates was impaired, certain serotypes, notably Infantis, Heidelberg, and Corvallis, showed a superior ability to maintain their growth rate. This finding is consistent with other studies that describe stress resistance in *Salmonella* as a highly diverse and strain-dependent trait, resulting from the selective pressure of the ecological niche within the poultry industry [33].

This diversity becomes even more critical when examining the response to oxidative stress mediated by sodium hypochlorite, a widely used disinfectant in the food industry. Although the Minimum Inhibitory Concentrations (MICs) for planktonic cells showed relative homogeneity among serotypes, the true adaptive advantage is revealed when evaluating sessile cells. Upon analyzing the MICB and the MBCB, we observed a drastic increase in tolerance. MICB values often doubled to 8 mM for most of the strong biofilm-forming isolates, and the MBCB reached values of 16 mM and even 32 mM in one *S.* Infantis isolate (Table 2 and Table 3). This represents a four- to eight-fold increase in the disinfectant concentration required to eradicate these biofilm-forming bacteria.

A direct link exists between the bacterial capacity to form a biofilm and the resistance to disinfectant effects. The results show that 123 out of 155 isolates (79%) are capable of forming biofilms, and of these, 52 (42%) are classified as “very strong” producers (Table 2). It is precisely these isolates that exhibit the highest MICB and MBCB values.

The discrepancy between planktonic susceptibility and biofilm tolerance highlights a critical flaw in current cleaning and disinfection paradigms. Industrial quality control regulations and protocols are often based on determining the MIC against bacteria in suspension [34]. Our data demonstrates that this approach is dangerously misleading as it overlooks important adaptive mechanisms guiding bacterial evolution. This creates a cycle of persistent contamination in which the problem is progressively aggravated, highlighting a fundamental gap between laboratory standards and the problems faced by industry.

Within the diverse *Salmonella* population analyzed, the *S*. Infantis serotype emerges as a particularly successful pathogen, exceptionally adapted to the poultry environment. Our data converge with findings from previous studies: this serotype presents the highest proportion of isolates with “very strong” biofilm-forming capacity (32 of 64 isolates, 50%), reaches high bactericidal concentrations in biofilms (up to 32 mM sodium hypochlorite), and exhibits the highest prevalence of MDR profiles (Figure 3) [19,35,36].

These results are consistent with the global epidemiological trend, which highlights the emergence of MDR *S.* Infantis as a high-risk clone predominantly associated with poultry production [37]. High levels of resistance to tetracycline, ampicillin, and trimethoprim-sulfamethoxazole (Figure 3 and Supplementary Table S2) are characteristic of the pattern conferred by the pESI (plasmid of emergent *S.* Infantis) megaplasmid. This plasmid, which was studied in previous work by our group and others [4,38], confers a resistance profile that reflects the global emergence of this serotype in the poultry industry worldwide [39,40,41,42,43].

The significance of the pESI plasmid is notable; this mobile genetic element not only carries many antibiotic resistance genes but also a collection of virulence and fitness factors that enhance biofilm formation, increase tolerance to acidic, osmotic, and oxidative stress, and improve environmental and host persistence [44]. Thus, this plasmid confers resistance to therapeutic antibiotics used in various poultry industries and strengthens biofilms, thereby providing tolerance to the chemical disinfectants employed on the production line.

This phenomenon creates a feedback loop throughout the entire production line. The use of antibiotics on farms to treat or prevent avian diseases exerts selective pressure that favors the proliferation of pESI-carrying strains. These same strains, upon arriving at the processing plant (slaughterhouse), are already equipped with a superior capacity to form biofilms, making them intrinsically more difficult to eradicate through standard sanitation processes. Furthermore, sublethal exposure to disinfectants in the plant can select for more robust biofilm producers, which may also carry MDR plasmids. This loop of selection and co-selection explains why *S.* Infantis has become such a successful and difficult-to-control pandemic clone worldwide [37].

While biofilm formation is a general survival strategy, our data revealed that certain stressors act as potent inducers, modulating the planktonic to sessile state transition. Exposure to osmotic stress triggered a significant increase in biofilm formation by the industrial isolates SE016 and SE081 (Figure 4). This effect contrasts with acid stress, which inhibited biofilm formation in all strains studied. This suggests that *S.* Infantis possesses specific and differential adaptive responses depending on the nature of the environmental challenge. This response has ecological relevance; osmotic stress is not just another stress condition, but a ubiquitous factor in poultry production lines. It is generated during processes such as brining and curing, and even more commonly, by the simple desiccation of work surfaces between cleaning and production shifts [45,46]. The low water activity on dry surfaces or in the presence of high solute concentrations imposes severe osmotic stress on bacterial cells. Therefore, the ability to respond to this stress by forming biofilms is a crucial survival mechanism. When observing the morphologies and architectures of these biofilms by scanning electron microscopy, the images revealed a direct correspondence with the biofilm biomass results.

Under osmotic and oxidative stress, the industrial isolates SE016 and SE081 showed a growth pattern of dense, mature biofilms. Cellular aggregates, high surface coverage, and complex architecture with cells embedded in an extracellular matrix were observed. These morphologies are consistent with findings from other studies on *Salmonella* biofilms [47]. In contrast, biofilms subjected to acidic stress showed a sparsely populated surface with isolated cells in small, disorganized groups, confirming the quantitatively observed inhibition.

Our results demonstrate that relevant environmental stressors, such as osmotic and oxidative stress, act as potent inducers of biofilm formation in industrial isolates of *S.* Infantis (SE016 and SE081). While our study did not delve into the molecular mechanisms, it is plausible that these observed phenotypes are due to the modulation of known regulatory networks in *Salmonella*. Biofilm formation is centrally controlled by the transcriptional regulator CsgD, which activates the production of curli fimbriae and cellulose, and by signaling through c-di-GMP, which promotes the transition to the sessile state [48,49]. It is possible that the increase in biomass and the polysaccharide-rich EPS matrix that we observed under osmotic stress is a consequence of the overactivation of these pathways.

However, a key limitation of our research is that we did not perform molecular or genetic analyses to determine the precise mechanisms of action in response to these environmental stressors. Future research, such as transcriptomic analyses, would be necessary to elucidate the specific molecular pathways affected by osmotic stress. Such studies could also determine the role of global stress regulators like RpoS, quorum-sensing systems like LuxS, or two-component systems like OmpR/EnvZ and PhoP/PhoQ, all known for their roles in persistence and biofilm formation [22,50,51].

The underlying mechanism for the increased resistance and biomass of stress-induced biofilms lies in the production of the EPS matrix. Industrial isolates SE016 and SE081 showed a significant increase in total EPS production under osmotic stress compared to the control condition and the non-industrial reference strain SARB27. This increase in EPS production is the direct quantitative explanation for the previously observed increase in biomass (Figure 4), demonstrating that *Salmonella* is metabolically active in the biofilm formation pathway in response to osmotic stress.

Analysis of the biochemical composition of this matrix showed that, under all conditions, polysaccharides constituted the major component, accounting for approximately 90% of the total EPS mass. This suggests that the adaptive response of *Salmonella* to osmotic stress is to increase the total production of EPS rather than altering its relative composition. This result is significant when considering the known functional roles of exopolysaccharides in *Salmonella*. Components like cellulose are key for structural integrity and have been shown to confer resistance to compounds such as hypochlorous acid (HOCl) [52]. Other polysaccharides, such as the O-antigen capsule and colanic acid, contribute to desiccation tolerance and protection against chemical stressors [53,54,55]. Collectively, this polysaccharide-rich matrix acts as a physical diffusion barrier, slowing the penetration of disinfectants, retaining water, and directly counteracting the effects of desiccation and osmotic stress [56].

The results of this study have profound implications for *Salmonella* control strategies in the poultry industry. We have demonstrated that stressors inherent to poultry processing, such as osmotic pressure and disinfectant exposure, not only fail to eradicate *Salmonella* but may also actively select for and strengthen the most problematic strains, such as pESI- and MDR-carrying *S.* Infantis.

It is important to acknowledge the limitations of our study to properly contextualize the findings. Our research was based on an in vitro model using plastic surfaces, which, although common, do not perfectly replicate the complex topology and physicochemical properties of all industrial materials and surfaces, a factor that can influence adhesion and biofilm formation differently [57]. Likewise, we focused on the most common environmental stressors, such as oxidative stress from sodium hypochlorite and the effects of pH variation and hyperosmolarity, although the industry utilizes a broader range of biocidal agents. Finally, these limitations open clear avenues for future research.

## 5. Conclusions

This study demonstrates that biofilm formation is the main adaptive strategy of *Salmonella enterica* in response to stress factors within the poultry production line. Among the 155 isolates analyzed, *S.* Infantis isolates stood out for their high resistance, strong biofilm-forming ability, and high prevalence of MDR. Inhibitory and bactericidal concentrations of sodium hypochlorite increased significantly when the bacteria were in a biofilm state, revealing a limitation in current disinfection practices. Osmotic stress was a strong inducer of biofilm and EPS production, especially in industrial strains carrying the pESI plasmid. The use of antibiotics and biocides in poultry farms creates an environment that fosters selection for pESI-carrying *S*. Infantis strains, which are primed for persistence throughout the entire production chain.

Importantly, this work suggests that monitoring programs must evolve from relying solely on planktonic susceptibility tests to assess biofilm-forming capacity, ideally under conditions that resemble the industrial environment. In addition, screening for markers like the pESI plasmid to identify high-risk clones will be of high importance.

Regarding biofilm control strategies, our data indicates that current sanitation protocols are insufficient. Future strategies should shift from a simple reliance on chemical agents towards integrated approaches that prevent biofilm attachment and maturation, and whose efficacy is validated against biofilms and their higher level of resistance compared to planktonic cells. Overall, the findings reveal a cycle of selection that favors the persistence of *S.* Infantis, highlighting the urgent need to revise industrial sanitation protocols to effectively address these resistant and highly adapted strains.

## Figures and Tables

**Table 1:** Distribution of the Minimum Inhibitory Concentration (MIC) of sodium hypochlorite among *Salmonella* serotypes.

	MIC [mM]			
Serotype	2	4	8	Total
Infantis	19	44	1	64
Corvallis	11	18	0	29
Senftenberg	6	18	0	24
Agona	0	18	0	18
Heidelberg	4	12	0	16
Typhimurium	0	4	0	4
**Total**	**40**	**114**	**1**	**155**

**Table 2:** Biofilm formation capacity and intensity among *Salmonella* isolates in sodium hypochlorite conditions.

	Biofilms Formation		Formation Intensity			
Serotype	Formers	No Formers	1	2	3	4
Infantis	57	7	4	12	9	32
Corvallis	19	10	6	3	2	8
Senftenberg	14	10	2	3	3	6
Agona	16	2	8	4	4	0
Heidelberg	10	6	4	2	0	4
Typhimurium	4	0	0	1	1	2
**Total**	**120**	**35**	**24**	**25**	**19**	**52**

**Table 3:** **Number of isolates under each value of Minimal.** Inhibitory (MICB) and Biocidal Concentrations for biofilm (MBCB) of sodium hypochlorite against established *Salmonella*.

	MICB		MBCB			
Serotypes	4 mM	8 mM	4 mM	8 mM	16 mM	32 mM
Infantis	8	24	0	21	2	1
Corvallis	5	3	2	5	1	0
Senftenberg	3	3	0	6	0	0
Agona	0	0	0	0	0	0
Heidelberg	4	0	1	3	0	0
Typhimurium	0	2	0	1	1	0
Total	20	32	3	36	4	1

## Data Availability

The original contributions presented in this study are included in the article and supplementary material. Further inquiries can be directed to the corresponding author.

## Supplementary Materials

The following supporting information can be downloaded at: https://www.mdpi.com/article/10.3390/microorganisms13081812/s1, Table S1: Physiological characteristics of Salmonella isolates: Maximum growth in culture media in response to osmotic and acid; Table S2: Antibiogram of Salmonella isolates and profile of multiresistance.
